# Clinical safety of combined therapy of immune checkpoint inhibitors and *Viscum album L.* therapy in patients with advanced or metastatic cancer

**DOI:** 10.1186/s12906-017-2045-0

**Published:** 2017-12-13

**Authors:** Anja Thronicke, Megan L. Steele, Christian Grah, Burkhard Matthes, Friedemann Schad

**Affiliations:** 1 Forschungsinstitut Havelhöhe gGmbH, Kladower Damm 221, 14089 Berlin, Germany; 2 Institute of Health and Biomedical Innovation, School of Public Health and Social Work, Queensland University of Technology, Park Road, Brisbane, VIC 4059 Australia; 3 Gemeinschaftskrankenhaus Havelhöhe, Kladower Damm 221, 14089 Berlin, Germany; 4 Klinikum Brandenburg der Medizinischen Hochschule Brandenburg, Hochstr 29, 14770 Brandenburg, Germany

**Keywords:** Targeted therapy, Immune checkpoint inhibitors, PD-1, CTLA-4, Mistletoe, *Viscum album* L., Drug interaction

## Abstract

**Background:**

Despite improvement of tumour response rates in patients with progressive and metastatic cancer, immune checkpoint inhibitors (ICM) induce toxicities in cancer patients. *Viscum album* L. (VA, mistletoe) extracts are applied as add-on cancer therapy especially in German speaking countries and within integrative and anthroposophical concepts with the goal to improve quality of life. The primary objective of this pilot observational cohort study was to determine the rate of adverse events (AE) related to ICM therapy with and without VA in patients with advanced or metastatic cancer in a certified Cancer Center.

**Methods:**

ICM or combined ICM/VA therapies were applied in patients with progressive or metastatic cancer. AE rates of both therapy groups were compared.

**Results:**

A total of sixteen cancer patients were treated with ICM: nivolumab (75%), ipilimumab (19%) or pembrolizumab (6%). The median age of the study population was 64 years (IQR 57.8; 69.3); 44% were male. Of the sixteen patients receiving ICM, nine patients received additional VA (56%; ICM/VA group) and seven did not (44%; ICM group). No statistically significant differences were seen between groups with respect to AE-rates (67% ICM/VA versus 71% ICM). Adjusted multivariate regression analysis revealed that concomitant application of VA did not alter the AE rate in ICM treated patients. 85% of AEs were expected ICM reactions. No AEs of grade 3 or greater were documented for the total study cohort.

**Conclusions:**

This is the first study evaluating the clinical safety profile of ICM in combination with VA in patients with advanced or metastatic cancer. The overall AE rate of the study cohort is comparable to AE rates of ICM treatment in the literature. Our data indicate a first impression that concomitant VA application may not alter ICM-induced AE rates. However, the nature of this study does not allow excluding possible immunological interactions between ICM and VA. Further prospective trials in larger study cohorts should focus on the assessment of safety aspects, clinical efficacy and health related quality of life in patients with combined ICM/VA therapy.

**Trial registration:**

DRKS00013335, retrospectively registered (November 27th, 2017) at the German Clinical Trials Register (www.drks.de).

## Background

Cancer cells are able to gain control over a number of inhibitory pathways that are important for controlling immune responses [1, 2]. By overexpressing programmed cell death protein ligands (PD-Ls) that bind to the immune checkpoint receptor programmed cell death protein 1 (PD-1), solid tumour cells can modulate T-cell activation in inflammatory cascades. Uncontrolled activation of the PD-1 receptor by cancer cells leads to anergy of antigen-specific T-cells and therefore diminishes their anti-tumour effectiveness. Blockade of the PD-1/PDL-1 pathway with immune checkpoint mononclonal antibodies (ICM) nivolumab (Obdivo®) and pembrolizumab (Keytruda®) can help to improve lung cancer treatment as shown by several clinical trials [3–6].

Another immune checkpoint is the cytotoxic T-lymphocyte-associated antigen-4 (CTLA-4) which is expressed on activated T-cells that modulates peripheral T-cell expansion after antigen presentation [7]. By inhibiting CTLA-4, CTLs are (re-)activated and able to sufficiently help to reduce tumour burden. Clinical phase III studies with CTLA-4 ICM ipilimumab (Yervoy®) in metastatic melanoma have shown superiority in survival over tumour vaccination [8, 9] and a survival benefit in combination with chemotherapy versus chemotherapy alone [10].

Regarding their toxicity profile, PD-1/PDL-1 and CTLA-4 immune checkpoint blockade has resulted in mild, severe and even fatal adverse events (AEs) [11, 12]. Grade 3–4 AE rates of up to 12% due to PD-1/PDL1 blockade [5, 13, 14] and up to 18% due to CTLA-4 blockade [8, 15] have been reported in cancer patients. Despite improved tumour response rates, the combination of anti-PD-1/PDL-1 and anti-CTLA-4 therapies also seems to potentiate grade 3–4 toxicities in cancer patients [9, 16]. In addition, potentiation in toxicities has already been seen with ICM in combination with chemotherapy [17]. Due to their new and elevated toxicity profile it is crucial to carefully monitor AEs related to ICM [18], especially in combination with other antineoplastic agents [18, 19]. Thus, the next era of immunotherapy will involve the search for safe combinatory anti-cancer agents which do not interfere with the PD-1/PDL-1 or CTLA-4 immunomodulatory mode of action and do not potentiate related toxicities.

*Viscum album L.* (VA), known as mistletoe, has a long traditional herbal history. It has effectively been utilized as an add-on therapy of cancer treatment in Europe, especially in German speaking countries and within integrative and anthroposophical concepts with the goal to improve quality of life [20–24]. Integrative therapies, comprising a systematic approach towards complementary alongside conventional therapies, intend to reduce physical and emotional symptoms and improve health-related quality of life (HRQL) in cancer patients [25]. The field of integrative oncology is growing combining patient-centred integrative and conventional therapies in the management of cancer diseases that are safe and effective [26]. The quality of published clinical VA studies has been criticized to vary considerably and according to a Cochrane review “*more high quality, independent clinical research is needed*” [20]. The survival benefit of VA in cancer is still a matter of controversial discussion [21, 22, 25, 27–29] mostly due to risks of bias and the heterogeneous quality of studies published so far. With regards to adverse effects the safety profile of VA has indicated that subcutaneous (s.c.) and intravenous (i.v.) applications of VA in cancer were safe with no serious AEs [30–33]. A meta-analysis of clinical studies that applied VA Iscador preparations concluded that it might have beneficial effects in psychosomatic self-regulation in addition to short-term quality of life-improving effects in cancer patients [34]. The experimentally assessed mode of action of VA comprises induction of apoptosis, inhibition of cell proliferation and angiogenesis of the tumour cells as well as anti-inflammatory and immunomodulatory effects [35–38]. Thus, the immunomodulatory nature of VA raises the question of whether there are immunologic interactions between VA and inhibitors of the PD-1/PDL-1 or CTLA-4 pathways that might lead to increased toxicity for cancer patients. On the other hand recent results of our group revealed that the adverse event rate of targeted therapy was significantly reduced in cancer patients when VA was concomitantly applied [30]. This is in line with the observation that VA is applied in integrative oncology concepts concomitantly to adjuvant antineoplastic treatment to improve tolerability of chemotherapy-induced toxicity [24, 39, 40]. We hypothesize that addition of VA therapy in the present study would either diminish or stabilize the known side effects of antineoplastic ICM treatment.

## Methods

### Study design

Safety and efficacy of anti-PD-1/PDL-1 and CTL-A4 ICM nivolumab, ipilimumab and pembrolizumab with or without concomitant VA therapy were examined in a pilot observational open cohort study. The primary outcome of the study was to investigate the occurrence of AEs during treatment with ICM with and without VA to assess the question of whether additional VA influences the AE rate in ICM treated patients with advanced or metastatic cancer. The secondary outcome was the explorative comparison of the disease response rate and overall survival in ICM and ICM/VA treated patients.

### Description of study participants

**Fig. 1:**
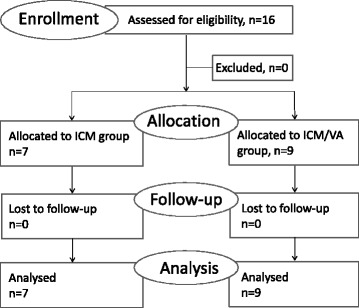
Flow chart of the study population. ICM, immune checkpoint monoclonal antibody; VA, *Viscum album* L., n, number of patients

The Hospital Havelhöhe (GKH) is a certified Cancer Centre demonstrating highest quality controlled cancer treatment and outcome for three organ classes (lung, breast and colorectal cancer) in accordance with certification guidelines of the Deutsche Krebsgesellschaft (DKG; German Cancer Association) and the Deutsche Krebshilfe (DKH; German Cancer Aid) [41]. Patients with advanced or metastatic cancer receiving ICM with or without concomitant VA therapy and being part of the Network Oncology (NO) registry were enrolled in the study between October 2013 and May 2016 (Fig. 1). Dates of first diagnosis for advanced and metastasized disease ranged from August 2008 until September 2015. The following patients were included: patients who were 18 years or older and of either gender with advanced or metastatic cancer, having given written informed consent, being registered in the NO and who received ICM with or without concomitant VA therapy. Patients were excluded if they had any of the following co-morbidities: active auto-immune diseases, symptomatic interstitial lung diseases, non-treated brain metastases, primary central nervous system-melanoma, human immune deficiency virus, hepatitis B, or hepatitis C. Written informed consent has been obtained. The Network Oncology (NO) registry study has been approved by the ethical committee of the Medical Association Berlin (Eth-27/10).

ICM treatment was i.v. applied according to the Summary of Product Characteristics (SmPC) [42–44]. VA therapy was applied s.c. according to SmPC. Off-label i.v. application was performed in individual cases. The rationale for VA application in patients of the current study was the improvement of HRQL and self-regulation in cancer patients by meliorating cancer and therapy related symptoms. I.v. or s.c. VA was administered at the discretion of the physician. Patients who agreed to the combined treatment were allocated to the ICM/VA group. The others were allocated to the ICM group.

### Sample size determination

A study cohort consisting of a total of twelve patients (ICM group: three patients, ICM-VA group: nine patients) would be needed to detect a significant increase in the relevant (immunologic) side effect with 80% power using an alpha value of 0.05. The expected mean difference (0.21) was derived from literature. The study had the power to detect large effects (Cohen’s d) with d [95 %CI] = 1.27 [−0.32, 2.86]. R-package “samplesize”, version 0.2–4 [45] was utilized.

### Data source and assessment

Demographic data as well as information on diagnosis, co-morbidities and previous treatment regimen were retrieved from the NO registry. NO is an accredited clinical registry of hospitals and out-patient practitioners specialised in IO [46]. Retrieved data included application procedures of ICM and VA therapy and related AEs. Date of death and last contact date were retrieved from the NO registry. Complete response, partial response (PR), progressive disease (PD) and stable disease (SD) were assessed according to revised RECIST guidelines, Version 1.1 for solid tumours [47].

AEs were classified as preferred terms according to the Medical Dictionary for Regulatory Activities (MedDRA®) Version 15.0 and grouped by System Organ Classes (SOC). AEs, serious AEs, adverse drug reaction (ADR) were designated according to ICH guidelines topic E2A [48]. Accordingly, an AE is “any untoward medical occurrence in a patient or clinical investigation subject administered a pharmaceutical product and which does not necessarily have to have a causal relationship with this treatment”. An ADR is defined as “a response to a drug which is noxious and unintended and which occurs at doses normally used in man for prophylaxis, diagnosis, or therapy of disease or for modification of physiological function” (41). In terms of severity, AEs were evaluated according to the Common Terminology Criteria for AEs (CTCAE) v4.03 [49] and designated as serious or non-serious according to ICH guidelines.

VA- or ICM-related AEs were immediately reported by physicians. If VA and ICM were given on the same day, VA was administered as the first infusion, before ICM treatment, thus, acute ADRs were attributable to VA treatment.

### Statistical methods

Univariate two-sided Fisher’s exact test was performed to detect differences in AE rates and tumour response rates between ICM and ICM/VA group. Multivariate regression analyses with binary outcome of experienced AE (yes/no) were performed to identify safety associated factors in ICM treated patients adjusting for age (in years), gender (male/female), VA treatment during ICM (yes/no), previous treatment with platinum-based chemotherapy (yes/no), with targeted therapy (yes/no), and with VA treatment (yes/no) and number of ICM-cycles greater than ten (yes/no). Continuous variables were described as median with interquartile range (IQR); categorical variables were summarised as frequencies and percentages. Data distributions were inspected graphically using box plots and histograms and were arithmetically examined for skewness. Stepwise backward variable selection with Akaike information criterion was performed for consideration of parameters within regression models. *P*-values <0.05 were considered to be significant. All statistical analyses were performed using the software R (Version 3.3.0) [50].

## Results

### Baseline characteristics

Sixteen cancer patients were treated with PD-1/PDL-1 or CTLA-4 ICM at GKH between October 2013 and May 2016. Table 1 shows the baseline characteristics of the patients. Of the sixteen patients 44% were male. The median age was 64 years (IQR: 57.8–69.3 years). Eleven patients had lung cancer (69%), four patients had a malignant melanoma (25%) and one patient had a pleural mesothelioma (6%). In twelve patients M1c-stage metastases were detected (75%). Predominant co-morbidities were respiratory disorders (10 patients, 62.5%), followed by blood & lymphatic disorders (6 patients, 37.3%), and cardiac disease disorders (5 patients, 31.3%), data not shown. Three of the ten patients with respiratory disorders had chronic obstructive pulmonary disease (COPD). Fourteen patients showed history of VA treatment (88%) (data not shown). Seven patients were pre-treated with at least two, and four patients with three or more anti-neoplastic systemic therapies including chemotherapy or targeted therapy. 50% of all patients received previous treatment with platinum-based chemotherapy, with the most frequently applied combination being carboplatin/vinorelbine (38%, *n* = 3), followed by cisplatin/vinorelbine and cisplatin monotherapy (each 25%, *n* = 2; data not shown). 13% (n = 2) of the study population were previously treated with targeted therapy (i.e. erlotinib or vemurafenib).

**Table 1:** Baseline characteristics of patients receiving ICM

Variable	Number of patients
Number of patients, n (%)	16 (100)
Age, years, median (IQR)	64 (57.8; 69.3)
Gender, male, n (%)	7 (43.8)
Primary tumour, n (%)	
Lung cancer	11 (68.8)
Malignant melanoma	4 (25)
Pleural mesothelioma	1 (6.3)
UICC stages, n (%)	
IIIA	4 (25.0)
IV	12 (75.0)

%, as percent from total patient number *n* = 16; *UICC*, Union for International Cancer Care; *VA*, Viscum album L.; *IQR*, interquartile range

### ICM therapy and combined ICM/VA therapy

Sixteen patients were treated with ICM therapy: twelve patients with nivolumab (75%), three patients with ipilimumab (19%), and one with pembrolizumab (6%). Of the sixteen patients, nine (56%) ICM-treated patients received VA extracts during the same interval while seven (44%) received no VA treatment during ICM treatment (Table 2). Of the ICM/VA treated patients, two received s.c. VA (22.3%), five s.c VA. (55.6%), and two patients received both i.v. and s.c. VA (22.3%). The median duration of VA therapy was 84 days with a range of 1–196 days of treatment. The median duration of ICM treatment was also 84 days with a range of 31–196 days (data not shown). One patient received percutaneous radiation for the treatment of metastases during the last day of ICM treatment (lung cancer). None of the sixteen patients were treated with chemotherapy or underwent surgery during ICM therapy.

**Table 2:** Dosage and application form of concomitant VA treatment during ICM therapy

VA treatment	Number of patients
VA, n (%)	9 (56.3)
* Abnobaviscum*, n (%)	7 (43.8)
* Abnobaviscum Fraxini,* 0.2 mg, s.c., n (%)	1 (6.3)
* Abnobaviscum Fraxini,* 20 mg, i.v., n (%)	1 (6.3)
* Abnobaviscum Fraxini,* 40 mg, i.v., n (%)	1 (6.3)
* Abnobaviscum Fraxini,* 60 mg, i.v., n (%)	1 (6.3)
* Abnobaviscum Fraxini,* 20–60 mg, i.v., n (%)	1 (6.3)
*Abnobaviscum Amygdali,* 0.2 mg, s.c. */Abnobaviscum Fraxini,* 20–40 mg, i.v., n (%)	2 (12.5)
* Helixor P*, 200 mg, i.v., n (%)	1 (6.3)
* Iscador Qu*, 5 mg, s.c., n (%)	1 (6.3)
No VA, n (%)	7 (56.3)

VA, Viscum album L.; %, as percent from total patient number *n* = 16; s.c., subcutaneous; i.v. intravenous

### Clinical safety of ICM versus combined ICM/VA treatment

Table 3 comprises all AEs that were documented during ICM or combined ICM/VA treatment. Each patient having experienced AEs has been designated with a letter (a-l; j was omitted).

**Table 3:** Frequency of AEs during treatment with ICM with or without VA therapy

System Order Class	AE (preferred term), grade 1–2	ICM (*n* = 7)	VA + ICM (*n* = 9)
Gastrointestinal	Constipation^1^		f
	Vomiting^4,^ ^1^		g
	Abdominal pain upper^1^	a, b	
	Abdominal colic^1^	c	
	Diarrhoea^1^	c	
	Nausea^4,^ ^1^	c	
General disorders and administration site	Decreased appetite^1^		f
	Pyrexia^1^	a	h
	Pain^1^		f
	Malaise^1^	c	f, h
Metabolism and nutrition disorder	Hyponatraemia^1^		i
	Marasmus		f
	Hypercholesterolemia^1^	d	
Musculoskeletal and connective tissue	Pain in extremity^1^		h
Nervous system	Altered visual depth perception^1^		h
Renal and urinary	Urinary tract infection^1^		f
Respiratory, thoracic and mediastinal	Bronchitis chronic		k
	Respiratory distress^1^		g
	Pneumonia staphylococcal^1^		g
	Bronchitis^1^	e	l
	Cough^1^	d	
	Pneumonia^1^	c	
Skin and subcutaneous	Decubitus ulcer		f
	Skin reaction^1^	c	i
Surgical and medical procedures	Supportive care		f
Total number of patients experiencing AE		5 (71.4^3^)	6 (66.7^2^)

Adverse events (AEs) were classified as MedDRA preferred terms and grouped by System Organ Class. Individual patients having experienced AEs during treatment are indicated by letters a-l (j was dismissed). ICM, immune checkpoint monoclonal antibody; VA, Viscum album L.; ^1^, expected according to ICM SmPC, ^2^ as percent from number of patients treated with ICM/VA (*n* = 9), ^3^ as percent from number of patients treated with ICM (*n* = 7), ^4^ adverse drug reaction, moderate

The total AE-frequency in the current study was 68.8% with eleven patients experiencing at least one AE. Two ADRs of moderate nature occurred in the study population. No grade 3 and 4 AEs (CTC, version 4.03) [49], no serious AEs, and serious adverse reactions (ICH) [48] were documented for the total study cohort. The most frequent (>10%) AEs in the current study were malaise (18.8%), pyrexia (12.5%), bronchitis (12.5%), and skin reaction (12.5%, Table 3).

With regards to treatment groups, five patients experienced at least one AE (71.4%) in the ICM group. One patient experienced one moderate ADR (14.3%), nausea, which was documented to be related to systemic ICM treatment. Further, in one patient (14.3%) ICM therapy (nivolumab) was interrupted for seven days due to bronchopneumonia, and symptoms were treated according to SmPC until disappearance. ICM therapy was then continued without re-appearance of pneumonia. Two patients that received ICM treatment experienced immune-related AEs (bronchitis: 14.3%, rash: 14.3%).

In comparison, of the nine patients treated with combined ICM/VA therapy, three did not experience any AE (33%). Six patients experienced at least one AE (66.7%), a rate which was 4% lower than the AE rate seen in the ICM group (not significant, *p* > 0.99). One patient treated with ICM/VA experienced one ADR (11%), nausea and vomiting. In this case the severity of the ADR was moderate and was indicated to be related to s.c. Abnobaviscum application. The VA therapy was discontinued and the patient recovered. Here the symptom lasted for one day only. In the ICM/VA group two patients experienced immune-related AEs (bronchitis: 11.1%, rash: 11.1%).

Adjusted multivariate regression analysis revealed no significant association between ICM/VA application and AE rate (OR: 1.467, 95%CI: 0.183–11.693 *p* = 0.720). Furthermore, we could show by adjusted multivariate analysis that the following variables, greater cycle-numbers (>10) of either ICM treatment alone or combined ICM/VA treatment, previous treatment with platinum-based chemotherapy, with targeted therapy or with VA were not associated with occurrence of AEs in the total patient cohort (data not shown).

### Clinical efficacy of ICM versus combined ICM/VA treatment

From sixteen patients nine patients showed progressive disease (56.3%), four showed stable disease (25.0%) and three patients showed partial disease response (18.8%, Table 4). In regards to treatment groups, progressive disease was observed in five patients treated with ICM (71.7%) and four patients (44.4%) treated with ICM/VA. In each treatment group two patients had stable disease (ICM group 28.6% versus ICM/VA group 22.2%). Partial response was only seen in patients treated with combined ICM/VA therapy (*n* = 3, 33.3%). No significant differences were detected between the groups. In the subgroup showing partial response, combined ICM/VA treatment resulted in stabilized size and number of metastases in one patient and size reduction of metastases and lymph nodes in two patients. In the cases of size reduction, one patient received nivolumab in combination with increasing doses of 20 to 60 mg i.v. Abnobaviscum and one patient received nivolumab in combination with 0.2 mg s.c. Abnobaviscum preparations.

**Table 4:** Tumour response after treatment with ICM with or without VA therapy

Disease response	ICM (*n* = 7)	ICM/VA (*n* = 9)	*p*-Value
Complete response, n (%)	0	0	NA
Partial response, n (%)	0	3 (33.3)	0.21
Stable disease, n (%)	2 (28.6)	2 (22.2)	NA
Progressive disease, n (%)	5 (71.4)	4 (44.4)	0.36

n, number of patients; ICM, immune checkpoint monoclonal antibody; *VA*, Viscum album L.%, as percent from number of total study group (*n* = 16); *NA*, not applicable

**Fig. 2:**
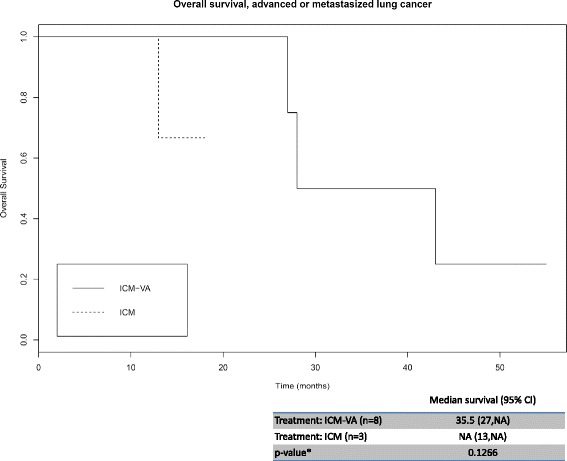
Kaplan-Meier curves by treatment arm; advanced or metastasized lung cancer. Kaplan–Meier curves for survival by treatment arm during total observation period (55 months, 4.6 years) and median survival in months for patients with advanced or metastasized lung cancer. ICM, immune checkpoint inhibitor nivolumab, ICM-VA, therapy with immune checkpoint inhibitors and concomitant *Viscum album* L.; CI, confidence interval. *based on log rank test

The Kaplan-Meier curves for overall survival (OS) are shown in Fig. 2 for a subgroup of eleven patients with advanced or metastasized lung cancer treated either with nivolumab (ICM) or nivolumab and VA (ICM/VA). Disease characteristics revealed squamous (45.5%), non-squamous (36.4%) and not specified NSCLC (18.2%). The median survival for all stage III/IV NSCLC patients combined was 28 months (95% CI: 27, NA), with a range of 13–43 months. By the end of the observational period five patients (31.3% of the subgroup) have died from cancer.

## Discussion

The results of the present study reveal that the overall AE rate (69%) experienced by the advanced or metastatic cancer study group was within the range or lower than the reported rate for AEs for nivolumab (60–78%), pembrolizumab (71–82%) and combination of ipilimumab/nivolumab (91–100%) [51]. Importantly, no grade 3 or 4 AEs according to CTC [49], no serious AEs as well as no serious adverse reactions according to ICH [48] were documented for the total study cohort. With regards to treatment groups, concomitant application of VA extracts did not alter the AE rate in ICM treated patients. Furthermore, neither s.c. nor off-label utilization of i.v. mistletoe application increased the AE rate in ICM treated patients in the current study. These results are in line with published tolerability of VA during chemotherapy and safety of i.v. and s.c. VA therapy [31, 32].

According to SmPCs the most frequently occurring published AEs (>10%) during therapy with ICM are fatigue, reduced appetite, nausea, diarrhoea, skin reactions, pruritus, vomiting, abdominal pain, urticaria, arthralgia, and malaise. The present study resembles this picture with respect to skin reactions and malaise as most frequent AEs.

With respect to immune-related (ir) adverse events, only ir-bronchitis (12.5%) and ir-skin reactions (12.5%) were documented in the whole present cohort. Recent literature reveals that approximately 25% of patients treated with ICM monotherapy experienced immune-related rash (all-grade). Whereas we observed an immune-related rash rate to be less than the published rates, the ir-bronchitis frequency in the present study cohort seems to be a log higher compared to literature: according to the SmPC of nivolumab, bronchitis rates are rather uncommon (up to 1 in 100 patients) and ir-pneumonitis rates account for around 3% of treated patients. However, in both bronchitis cases, the patients already showed signs of pulmonary comorbidity. These were a cough in one patient and pneumonia in the other. The immune-related symptoms resolved without the necessity of hospitalization, of radiologic, endoscopic, or operative intervention, of permanent discontinuation of treatment or application of high-dose corticosteroids. Interestingly, the observed ir-rash frequency in the total study cohort was one to two logs higher than the published ir-rash frequency of intravenous (2%) or subcutaneous (0.4%) VA treatment in cancer patients [31, 32]. However, as ir-rash frequencies are comparable between the ICM-VA and ICM group in the present study we assume that these immune-related skin reactions rather derive from ICM treatment itself. With regards to comparability of irAEs between both groups we further assume that the irAE rate was not altered due to additional VA treatment.

Most AEs (85%) observed in the present study were expected AEs according to ICM SmPC (e.g. pyrexia and malaise); however, unexpected AEs were chronic bronchitis, *decubitus ulcer* and marasmus seen in two patients. The chronic bronchitis may have been related to the COPD status in one patient. Marasmus and *decubitus ulcer* were seen in the other patient that was treated with 180 mg Ipilimumab and 200 mg i.v. Helixor P and may be explained by the patient’s reduced general condition described by being bedridden, experiencing obstipation, reduced appetite and needing to be taken care of.

Overall survival analysis was performed but due to the small numbers of patients, no definitive conclusions can be drawn from these data for a comparison of beneficial effects on survival. The present results regarding tumour progression status are, to our knowledge, the first preliminary clinical data for combined VA/ICM treatment. At the date of analysis, none of the study subjects had achieved complete disease response. While complete response has been reported in ICM studies (0.7% for nivolumab and 2–3% for pembrolizumab [44]), we suspect that the absence of patients showing complete response in our study is related to the small sample size. The disease response rates in the present study were in line with published ICM data [42, 44]. Interestingly, partial remission was seen only in patients treated with the combined VA/ICM therapy regimen, therefore the possibility of synergistic antineoplastic effects between VA and ICM cannot be excluded. However, no final conclusions are to be drawn regarding the influence of combined treatment on disease response in the present study due to limited sample size.

Besides inhibiting proliferation and inducing apoptosis [38] VA compounds lectins and lipophilic VA triterpenes have been shown to possess immunomodulatory properties in vitro [52, 53] and in vivo [54], inhibit proliferation and induce apoptosis in breast carcinoma cells in vitro*.* VA lectins (VAL) increase expression of co-stimulatory molecules in blood derived dendritic cells in a Toll-like receptor 4 dependent way [52]. They increase the percentage of Human Leukocyte Antigen-antigen-D-Related-positive T lymphocytes in vitro [52]. Incubation of peripheral blood mononuclear cells with VAL enhances the expression and secretion of a plethora of cytokines [53]. In vivo studies revealed IL-12 dependent activation of natural killer cells by VAL [55]. These immunomodulatory properties, in addition to the associated sound safety profiles [31, 32], observed improvements in HRQL and self-regulation [34, 39, 56, 57] make VA extracts a potentially attractive add-on therapy in antineoplastic concepts. Clinical trials assessing the addition of VA applications to conventional treatment regimens have shown that VA can reduce chemotherapy ADRs and improve tolerability of chemotherapy [24, 39]. The present study did not show significant reduction in cancer or ICM-related AEs by additional VA treatment, possibly due to the small number of patients enrolled and the limited observation duration. Furthermore, due to the nature of the current study, long-term side effects could not be determined. Nevertheless, this pilot study provides a first impression on the safety aspects of concomitant VA/ICM treatment in patients with advanced or metastatic cancer. Nevertheless, relevant pharmacological interferences between the known immunomodulatory mechanisms of VA and ICM therapy need to be investigated.

## Conclusions

The present pilot study gives a first insight into application of new generation immuno-oncological treatment with the natural immune-stimulant VA in patients with advanced or metastatic cancer. The overall AE rate for ICM treatment observed in the current study was in line with previously published studies. Furthermore, our data indicate a first impression that i.v. and s.c. applications of VA extracts during therapy with ICM may not alter ICM-induced AE rate, and that immune-related AE frequency was balanced in the ICM and ICM/VA group. However, the nature of the present study does not allow excluding possible immunological interactions between ICM and VA. In the light of newly approved cancer indications in the field of immuno-oncology, further studies in larger patient cohorts and respective experimental designs are warranted to assess immunological interactions between these two immunological active substances, clinical efficacy and HRQL in cancer patients receiving combined ICM/VA therapy.

## Abbreviations

ADR: adverse drug reaction
AE: adverse event
CD4+/Th1: cluster-of-differentiation/t-helper cell-1
COPD: chronic obstructive pulmonary disease
DKG: German Cancer Society
DKH: German Cancer Aid
i.v.: intravenous
ICH: International Council for Harmonisation of Technical Requirements for Pharmaceuticals for Human Use
ICM: immune checkpoint monoclonal antibodies
IO: integrative oncology
IQR: interquartile range
MedDRA: Medical Dictionary for Regulatory Activities
NO: network oncology
NPI: non-pharmacological intervention
PD-1: programmed cell death protein −1
PDL-1: PD-1 ligand
s.c.: subcutaneous
SmPC: Summary of Product Characteristics
UICC: Union for International Cancer Care
VA: *Viscum album* L
